# Characterization and Validation of a New 3D Printing Ink for Reducing Therapeutic Gap in Pediatrics through Individualized Medicines

**DOI:** 10.3390/pharmaceutics15061642

**Published:** 2023-06-02

**Authors:** Eduardo Díaz-Torres, Javier Suárez-González, Cecilia N. Monzón-Rodríguez, Ana Santoveña-Estévez, José B. Fariña

**Affiliations:** 1 Departamento de Ingeniería Química y Tecnología Farmacéutica, Campus de Anchieta, Universidad de La Laguna (ULL), 38200 La Laguna, Tenerife, Spain; ediaztor@ull.edu.es (E.D.-T.); cmonzonr@ull.edu.es (C.N.M.-R.); jbfarina@ull.edu.es (J.B.F.); 2 Instituto Universitario de Enfermedades Tropicales y Salud Pública de Canarias, Universidad de La Laguna (ULL), Avenida Astrofísico Francisco Sánchez, s/n., 38200 La Laguna, Tenerife, Spain; 3 Programa de Doctorado en Ciencias Médicas y Farmacéuticas, Desarrollo y Calidad de Vida, Universidad de La Laguna, 38200 La Laguna, Tenerife, Spain

**Keywords:** 3D printing, individualized medicines, quality by design (QbD), printlets, PAT, semi-solid, extrusion, SSE

## Abstract

3D printing technology can be used to develop individualized medicines in hospitals and pharmacies, allowing a high degree of personalization and the possibility to adjust the dose of the API based on the quantity of material extruded. The main goal of incorporating this technology is to have a stock of API-load print cartridges that could be used at different storage times and for different patients. However, it is necessary to study the extrudability, stability, and buildability of these print cartridges during storage time. A paste-like formulation containing hydrochlorothiazide as a model drug was prepared and distributed in five print cartridges, each of which was studied for different storage times (0 h–72 h) and conditions, for repeated use on different days. For each print cartridge, an extrudability analysis was performed, and subsequently, 100 unit forms of 10 mg hydrochlorothiazide were printed. Finally, various dosage units containing different doses were printed, taking into account the optimized printing parameters based on the results of the extrudability analysis carried out previously. An appropriate methodology for the rapid development of appropriate SSE 3DP inks for pediatrics was established and evaluated. The extrudability analysis and several parameters allowed the detection of changes in the mechanical behavior of the printing inks, the pressure interval of the steady flow, and the selection of the volume of ink to be extruded to obtain each of the required doses. The print cartridges were stable for up to 72 h after processing, and orodispersible printlets containing 6 mg to 24 mg of hydrochlorothiazide can be produced using the same print cartridge and during the same printing process with guaranteed content and chemical stability. The proposed workflow for the development of new printing inks containing APIs will allow the optimization of feedstock material and human resources in pharmacy or hospital pharmacy services, thus speeding up their development and reducing costs.

## 1. Introduction

The development of individualized medicines in hospitals, pharmacy services, and pharmacies is beneficial in closing the therapeutic gap within the pediatric population. In this sense, this procedure provides the opportunity to formulate the active pharmaceutical ingredient (API) in a medicine that is individualized to a certain patient according to, for example, ability to swallow, intolerance to some excipients, preference for a certain taste or mouthfeel, etc. A high degree of personalization is possible using 3D printing technology, as excipients can be added to provide a specific taste or color, and the shape or size of the dosage form can be modified to accommodate, if necessary, the patient’s ability to swallow, taste, acceptability, etc. The most important feature, however, is the ability to adjust the dose based on the material extruded, similar to traditional liquid formulations, but offering increased stability, similar to solid formulations [1]. In this regard, healthcare professionals have a good understanding of the benefits and possibilities of 3D printing for the development of individualized medicines [2,3]. Such is the interest in this opportunity that clinical studies have already been made for the administration of chewable printlets for treating Maple Syrup Urine Disease in pediatrics [4,5]. Semi-solid extrusion (SSE) is one of the multiple 3D printing technologies available in the market. Generally speaking, this technique involves creating a semi-solid gel or paste (printing ink) with great extrudability, flowability, and buildability, depositing the material, solidifying it, and ending with solvent evaporation and solidification until getting the final dosage form, called a printlet [6,7]. In comparison to other 3D printing processes, SSE offers the most potential for usage in health-related situations (hospitals or pharmacies) because of lower costs and the fact that higher temperatures, which can degrade the API, are not required to produce dosage forms. Pre-filled and disposable materials can be used; thus, cross-contamination is reduced, and safety is ensured [7]. The main goal of incorporating this technology in hospitals and pharmacies would be to have a stock of printing inks of various APIs that could be used at different storage times and for different patients, as doses could be modified based on the amount of material extruded in each dosage form. However, it is essential to ensure the printability and stability of the print cartridge, which consists of the primary packaging (e.g., syringe) and the paste-like printing ink containing the API, during storage and multiple extrusion cycles. For this purpose, it is necessary to study their rheological behavior. Paste and gel-like materials have already been analyzed for 3D printing using an oscillating rheometer and texturometer, but it has been concluded that data from the last study were more accurate in predicting the printing pressures of pastes due to the better analogy to SSE printing conditions [8,9,10,11,12]. Therefore, the extrudability profiles will provide essential information, such as extrusion pressure, yield stress, steady flow, etc., which will determine the extrudability and flowability of the paste and, lastly, the quality of the printlets. However, changes in these parameters have not been studied during storage or in normal conditions in a pharmacy service, where multiple extrusion cycles could be performed with the same print cartridge [12].

One API for which a print cartridge is required is hydrochlorothiazide (HCT), a diuretic API used to treat hypertension, edema, idiopathic renal hypercalciuria, and insipid diabetes with a dosage of 1–2 mg/kg. Since there are currently no commercially available child-friendly formulations that take into account the World Health Organization’s (WHO) Child Growth Standards, printlets with doses ranging from 6 mg to 24 mg will be necessary and, consequently, newborn and children up to 8 years old could be treated [13,14]. Studies related to dose adjustments have been carried out, taking into account the number of layers deposited at the printing platform [1,15]. However, this procedure has limitations, as the dose of API is restricted to the amount of API per layer. Therefore, the possibility of small dose adjustments is limited. For example, only dose multiples of 2 could be obtained if 2 mg of API were applied layer by layer. In addition, the ability to adjust a wider range of doses would be beneficial to print different patient prescriptions (same API/different doses) in the same printing process, saving time, materials, and resources. In terms of quality by test (QbT) evaluation of these printlets, international pharmacopeias have not published recommendations in this sense, but, at the very least, mass and content uniformity must be ensured. In order to study the API content in each dosage form, tests such as the uniformity of dosage units or uniformity of content of single-dose preparations should be performed [16,17]. However, this procedure means the analysis of up to 30 printlets, which would require the preparation of specific batches in pharmacies and hospital pharmacy services for quality control.

For this reason, a pressure sensor control that can characterize the printing ink and have perfect control over the pressure applied during the whole printing process, as well as the ability to detect printlets that do not meet quality standards, has just been added to SSE 3D printing platforms [7]. This is a component of the process analytical technology (PAT) strategy that enables in-line manufacturing process control with the ability to identify dose units with poor quality features [18]. In addition, other analytical techniques could be included as part of the PAT. For example, computer vision to determine the area and perimeter of the printlet as indicative of the buildability of the printing ink, the surface reduction as a drying check parameter of the printlet, or the printability of square pores in the case of scaffolds [19,20,21,22]. The incorporation of this strategy opens the door to a more efficient, cost-effective, and high-quality 3D printing, particularly for incorporation into pharmacy services, because fewer QbT would be required. Moreover, in the future, no unit will be wasted in performing such tests. This will allow a decentralized manufacturing process to be put in place without giving up a unified quality control that allows the production of dosage forms with the required final quality attributes (CQA) [23].

Taking into account everything mentioned above, this study aims to provide a valid methodology for the rapid development of appropriate SSE 3DP inks for pediatrics, containing HCT as a model API and evaluate changes in the extrudability profile during storage or after multiple extrusion cycles, in order to explain and address any issues that might develop following application in hospitals or pharmacy services. In addition, the possibility of printing several dosage units with different doses was also studied, as well as the stability of these printlets once elaborated. Finally, in-line measurements of pressure and computer vision were taken within the process to control it as part of the strategy to reduce QbT on behalf of the PAT.

## 2. Materials and Methods

### 2.1. Materials

Hydrochlorothiazide (HCT) and polyvinylpyrrolidone (PVP) were provided by Acofarma^®^ (Madrid, Spain). Ac-Di-Sol^®^ (Croscarmellose Sodium) was kindly provided by IMCD España (Barcelona, Spain). Lactose monohydrate was purchased from Sigma-Aldrich (Burlington, MA, USA). A FabRx M3dimaker printing platform (London, UK) equipped with a pressure instrumentalized SSE motor-driven printhead (Laguna SSE printhead, FabRx Ltd., London, UK) was used to elaborate printlets. Injekt™ Syringe 20 mL Luer Lock (B. Braun Medical Inc. OEM, Melsungen, Germany) with Fisnar QuantX™ 20 ga Pink nozzles (Fisnar, Glasgow, UK) was also used.

### 2.2. Three-Dimensional Printing Ink Preparation

HCT was selected as a model drug to be incorporated into the printing ink as it has already been studied by the research team [24]. To prepare the printing ink, all the components were milled separately and added to a Cito Unguator B/R mixer jar (Microcaya, Bilbao, Spain) in the following order: 41.7% HCT, 25.0% lactose monohydrate, 8.3% PVP, and 25.0% Ac-Di-Sol^®^. The formulation was mixed for 1.5 min at 650 rpm, and purified water was added in order to obtain a homogeneous paste-like formulation [7]. Then, using a spatula, the mixture was transferred to the print cartridge (syringe).

### 2.3. Design of the Printlets and Setting the Printing Parameters

#### 2.3.1. Design of the 3D Shapes

Autodesk Fusion 360 (ver. 2.0.9011, Autodesk Inc., San Rafael, CA, USA) was used to design the desired cylindrical printlets for each study. The 3D model was exported as a stereolithography file (.stl) into the 3D printing software, Slic3r (GNU Affero General Public License, ver. 3.0).

#### 2.3.2. Slic3r Profiles (Printing Settings)

The printing settings were chosen as follows to obtain high-quality printlets and brief printing time: printing surface material was polypropylene; printing speed was 7 mm/s; first layer thickness was 0.4 mm; and successive width was 0.6 mm. Finally, printlets were stored on the printing platform for 6 h in order to dry, keeping a complete record of temperature and humidity, 25.5 ± 0.04 °C and 63.2 ± 0.4%, respectively.

### 2.4. Rheological Characterization of the Printing Ink

The rheological behavior of the ink was analyzed before printing using a rheometer (Malvern Instruments Inc., CVO 100 Rheometer, Malvern, UK) suited with an HPTD 200 Peltier hood and a disposable aluminum plate of 20 mm in diameter. The analysis was performed in triplicate at 40 °C and 0.6 mm of gap. A frequency sweep was performed with a shear rate of 50 Pa and a frequency from 0.01 Hz to 100 Hz. The critical strain of the paste was obtained from the amplitude sweep, which was performed from 0 to 10 KPa and a frequency of 1 Hz. Finally, a creep recovery test was performed to evaluate the viscoelastic properties of the ink at a shear stress of 500 Pa and 60 s of creep time. The balance between the loss (G″) and the storage (G′) modulus has been studied using the parameter tan δ (G″/G′). Data collection was performed using Bohlin software (ver. R6.51.0.3, Malvern Instruments, Worcestershire, UK).

### 2.5. Stability Evaluation of the Printing Ink

#### 2.5.1. Extrudability Analysis of the Printing Ink

In order to study the extrudability profile, a total amount of 50 mL of the printing ink was prepared and distributed into five different Braun Injekt™ syringes of 20 mL with luer lock. The extrusion profile was defined using the parameters set out in Table 1.

**Figure 1:**
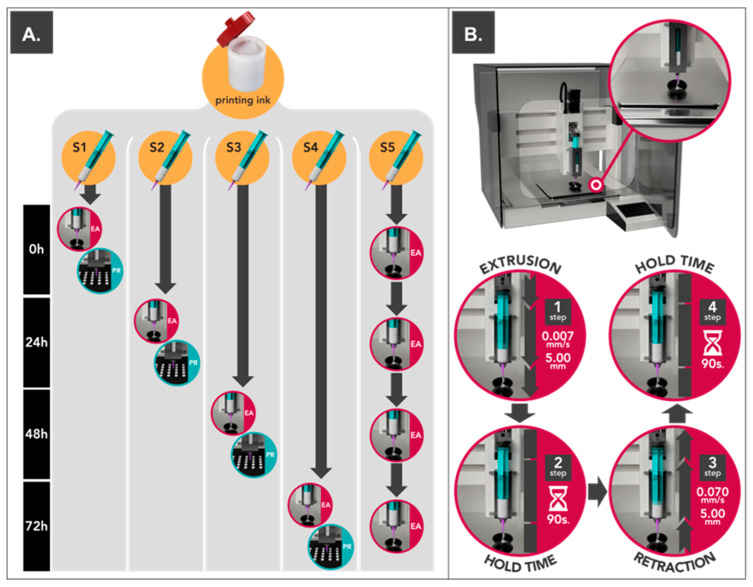
(**A**) Schematic representation of the stability evaluation of the print cartridge: extrudability analysis (EA) and printing process (PR). (**B**) Consecutive steps performed in each extrusion/retraction cycle during extrudability analysis. Detailed view of the installed scale over the 3D printing building plate.

Each of the print cartridges was studied under different conditions (Figure 1A). On the one hand, four print cartridges were stored at 25 °C and 60% of relative humidity (JP Selecta™ Medilow Refrigerated Cabinet) for being extruded at 0 h (S1), 24 h (S2), 48 h (S3), and 72 h (S4) to study changes within storage time (Figure 1A). On the other hand, the remaining print cartridge (S5) was extruded at 0, 24, 48, and 72 h in order to evaluate possible changes after being exposed to repeated extrusion cycles over time (Figure 1A). All print cartridges (S1–S5) were hermetically sealed using luer lock caps to prevent water evaporation.

Tests were performed using a pharmaceutical 3D printing platform (M3DIMAKER, FabRx Ltd., London, UK) equipped with the innovative pressure instrumentalized SSE motor-driven printhead (Laguna SSE printhead, FabRx Ltd., London, UK) using 0.61 mm (20 ga.) conical nozzle, a flow of 0.007 mm/s, and 40 °C. Texturometer Utility for M3DIMAKER (FabRx Ltd., London, UK) made it possible to execute extrusion/retraction cycles; this consisted in applying a downward force on the plunger of the print cartridge for a distance of 5 mm and at 0.007 mm/s of displacement speed, followed by a hold time of 90 s and a retraction of the same distance previously performed (with a displacement speed of 0.070 mm/s). The final step consists of a holding time of 90 s (Figure 1B). These variables were selected in order to simulate the 3D printing process used to elaborate printlets in the following sections [7,8,9,10]. During the whole cycle, the accumulated weight of the extruded semi-solid material was registered using a precision scale ENTIRIS153I-1S (d = 0.001 g) (Sartorius, Germany) placed over the 3D printing building plate as shown in Figure 1B.

#### 2.5.2. Printability of the Printing Ink

As shown in Figure 1A, once the printing ink was characterized, a batch of 100 printlets with 10 mg of HCT per dosage unit was elaborated for each print cartridge (S1–S4). Then, the quality of the printlets was evaluated, taking into account their general aspect and content of API, expressed as % of the declared value (DV). The same g-code file was used for each printing process.

For this purpose, the weight of each printlet was determined using an Analytical Balance XSR105DU/M (d = 0.01 mg) (Mettler Toledo, Greifensee, Switzerland), and the contents of three randomly selected printlets were determined using an Ultra Performance Liquid Chromatography system (UPLC) after drying. Analytical separations were made in an Acquity UPLC^®^ H-Class using a previously validated analytical method [24,25]. In order to analyze the content of API, each printlet was placed in a 25 mL flask with 10 mL of purified water and placed in an ultrasonic bath for 8 min. Then, 10 mL of methanol was added to dissolve the API, and the volume was completed with purified water. Finally, the samples were filtered using 110 mm filter paper (Albet LabScience, Barcelona, Spain) and diluted with mobile phase up to 10 μg/mL to be analyzed in the UPLC system. For the purpose of ensuring the optimum performance of the system, a 10 μg/mL pure pattern of HCT was analyzed daily [24].

The values in the data set below 25% and 75% of the complete data points (first and third quartile, respectively), median, mean, standard deviation, etc., were calculated. Tukey’s test was applied to determine the outliers for the values of mean applied pressure, total area under the curve (AUC) per printlet, and weight of printlet in order to study their variability as possible critical process parameters. Particularly, 7.5% of average was used as limits in the case of weight variability, being stricter than the limits collected in the pharmacopeia for this weight in the case of tablets, 10% [26].

### 2.6. Stability Evaluation of the Printlets

Using the printing ink from S1, a single batch of 100 printlets with 10 mg of HCT was elaborated under the same conditions mentioned in the design of the printlets and setting of the printing parameters section. Once the printlets were completely dry through 6 h of drying time at room temperature, they were weighed and stored under accelerated conditions according to The International Council for Harmonisation of Technical Requirements for Pharmaceuticals for Human Use (ICH) (ICH 110L, Memmert, Schwabach, Germany) [27]. Then, printlets were analyzed in triplicate at 0, 7, 14, 21, and 28 days, and the content of API was expressed as % DV.

### 2.7. Evaluation of the Ink and Process Capability to Print Different Doses

**Figure 2:**
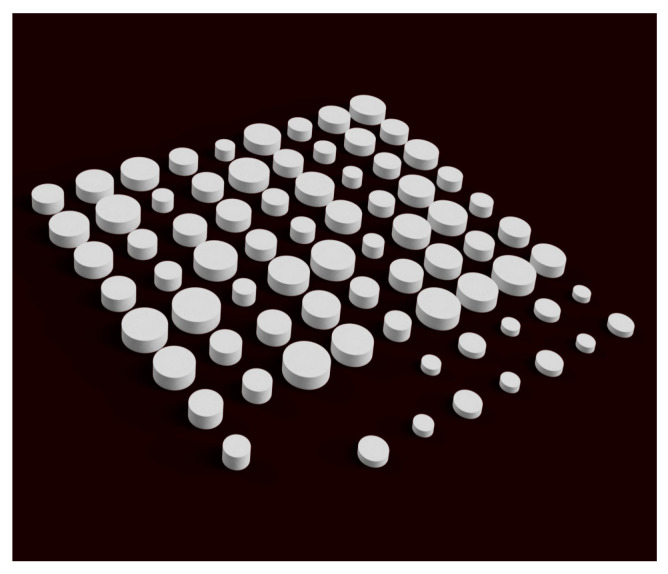
Rendered 3D model of the printlets with different doses randomly distributed.

Printlets from 2 to 24 mg of HCT were designed, taking into account the volume of mass extruded required to obtain such doses as well as the desired height, 1.2 mm or 2.4 mm. Each printlet model, corresponding with one dose, was duplicated four times, and all were randomly distributed in the design space, as can be seen in Figure 2.

Each printlet was weighed on an Analytical Balance XSR105DU/M (d = 0.01 mg) (Mettler Toledo, Greifensee, Switzerland) and analyzed in triplicate (as mentioned in the previous section). The analysis of variance (ANOVA) was carried out to confirm the linearity of the relation between the theoretical and experimental doses. Results were expressed as % DV and ±10% of DV was established as limits.

### 2.8. In-Line Process Control

In-line measurements were taken as part of the PAT strategy analyzing pressure and computer vision data. The information collected by the sensors installed on the printing platform was analyzed by a Python algorithm designed by the research group. Based on the values of average applied pressure, AUC per printlet, computer vision, using the library of computer vision OpenCV (open-source Apache 2 License), and outlier trends in pressure and AUC throughout the printing process, it was possible to determine which dosing units would not meet the critical quality attribute (printlet weight).

#### 2.8.1. Extrusion Pressure Control

The applied pressure values of the Laguna SSE printhead were collected using the built-in data logger of the M3DIMAKER 3D printing platform to ensure that the process remained under control throughout the entire production of the printlets and to look out for any potential obstructions or the presence of air that might have an impact on the final CQA of the dosage forms. The recorded pressure values throughout the manufacture of the batch of dosage forms were represented in 3D scatter form for each coordinate in space (X, Y, and Z).

#### 2.8.2. Computer Vision

Dimension and theoretical equivalent diameter were obtained by g-code file and compared once the printing process was completed and dry, expressed as shape fidelity (1) and size reduction (2), respectively. Computer vision was also used for printing error detection. Images to be analyzed were taken using the telephoto camera of the iPhone 14 Pro from Apple (Cupertino, CA, USA).

(1) $$Shape\;fidelity=\frac{Theoretical\;equivalent\;diameter}{Equivalent\;diameter,t0\;h}\cdot 100$$

(2) $$Size\;reduction=\left(1-\frac{Equivalent\;diameter,t6\;h}{Equivalent\;diameter,t0\;h}\right)\cdot 100$$

## 3. Results and Discussions

### 3.1. Rheological Characteristics of the Printing Ink

**Figure 3:**
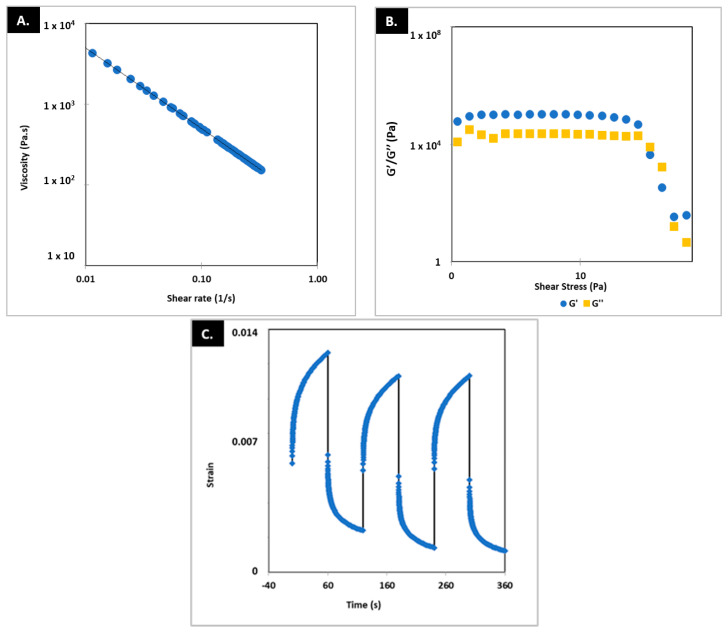
Results for frequency (**A**), amplitude (**B**), and creep recovery test (**C**). G′: storage modulus. G″: loss modulus.

Figure 3 shows the results of the rheological characterization of the printing ink. The tests carried out (frequency and amplitude sweep) demonstrated the viscoelastic behavior of the printing ink. Additionally, the creep recovery test demonstrated a rapid recovery of rheological properties when the stress conditions to which they were subjected during the printing process ceased. However, a complete recovery was not achieved in the first cycle. The aptitude of the printing ink to be used in 3D printing is worth noting as it showed high viscosity during rest (tan δ < 1), a drop under reasonable shear stress, >1000 Pa, and rapid recovery of the viscosity once it is deposited on the printing platform (shear stress = 0), particularly under this high shear stress tan δ > 1, which means it favors flow and formation of section-stable strands at the nozzle tip [10].

### 3.2. Stability Evaluation of the Printing Ink

#### 3.2.1. Extrudability Analysis

**Figure 4:**
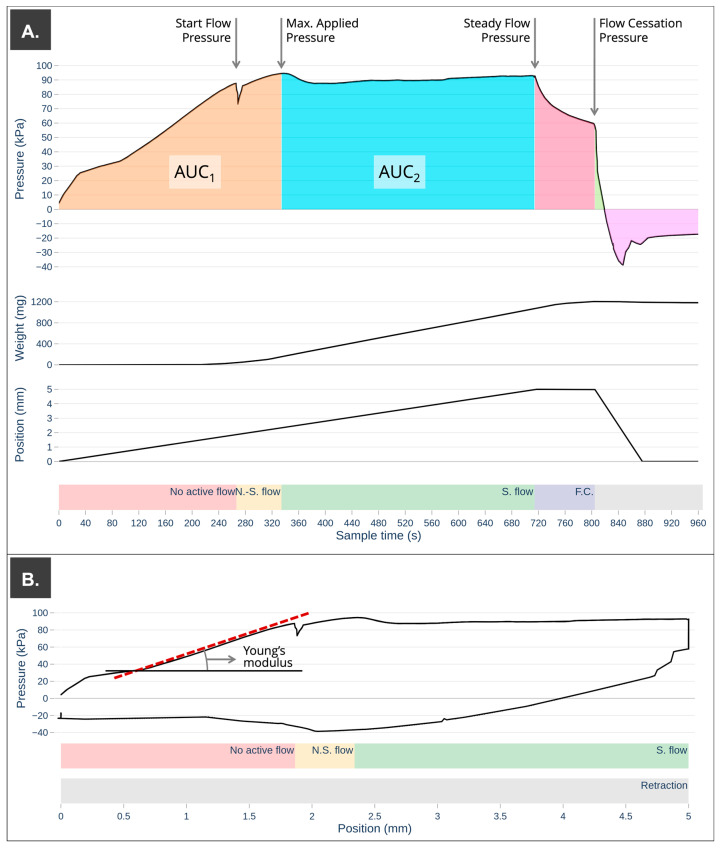
Extrudability analysis for S5 print cartridge at 0 h of storage time where the colored backgrounds highlight the most important moments of the extrusion process: N.S. flow: non-steady flow, S. flow: steady flow, and F.C.: flow cessation. (**A**) Pressure–time plot with values of particular relevance (yield stress, max. applied pressure, steady flow pressure, and flow cessation pressure) and areas under the curve (AUC) calculated as a function of time. (**B**) Young’s modulus calculation from pressure–position plot.

A graphical representation of the extrudability profile is shown in Figure 4.

The first step of the extrudability analysis consisted of a downward displacement of 5 mm at a constant speed of 0.007 mm/s. As expected, the first stage of mass compression occurred [9,12,20]. A progressive increase in the recorded applied pressure was observed, but no semi-solid mass came out through the nozzle. After this period of time, the system acquired enough pressure, and the printing ink began to flow through the nozzle (determined as a weight recorded on the scale > 1 mg) (Figure 4). The minimum stress required to induce flow is known as the yield stress or yield point [20,28,29,30,31]. Moreover, this stress indicates the limit of elastic behavior. According to some authors, the yield stress can be used to indirectly determine how accurately and faithfully a print will turn out, as well as how well the ink will support additional stacked layers.

The results of the extrudability analysis are shown in Table 2. The pressure value was shown to be in the same order of magnitude for all the printing inks tested except for those studied at 24 h of storage time, where it was slightly lower, although this value was recovered after 48 h. Several authors suggest that high-yield stresses in which viscosity decreases rapidly result in adequate extrudability and higher printing accuracy [20,32].

Although many authors highlight that the determination of the yield point allows determining the pressure necessary to extrude a semi-solid material through a nozzle, other authors do not consider this parameter alone to be a good indicator of printability, so other parameters have been studied in this test [20,32].

Next, a non-stationary flow phase was observed in which a non-linear increase in the accumulated extruded mass was recorded, and the applied pressure continued to increase progressively until a maximum pressure value was reached, at which the stationary flow phase began for each of the samples analyzed. From this maximum applied pressure, a slight decrease in pressure occurred, reaching a constant value of steady flow pressure. Differences in the maximum applied pressure were observed between printing ink; this increased with controlled storage time. On the other hand, it was observed that the formulation that was subjected to several extrusion–retraction cycles (S5) showed a higher value of maximum applied pressure compared to the one that was extruded only once for the same storage time (S1–S4), increasing this difference in storage times greater than 48 h. This increase in the value of the maximum applied pressure can affect the mass variation of the initial printlets. If the initial printing pressure is not set close to this pressure value, the stationary flow of semi-solid material through the nozzle is not achieved until a certain amount of ink is extruded.

From this point on in the extrudability analysis, the downward movement of the plunger produced a steady flow of material through the nozzle. Similar to what was observed for the recorded values of maximum applied pressure, the steady flow pressure increased as the storage time increased. It seems that the application of a repeated extrusion pressure (S5) causes an increase in the steady flow pressure compared to that which was characterized for the first time at each of the established times. This difference increases over time to more than 16% (S3–48 h vs. S5–48 h) and 23% (S4–72 h vs. S5–72 h). The fact of an increase in the maximum applied pressure and the steady flow pressure must be taken into account when using the same print cartridge on different days by setting a validity period for the print cartridge or by setting an increase in the start pressure to guarantee a steady flow during the whole printing process.

The progressive increase in the maximum applied pressure and the steady flow pressure with increasing storage time could be due to a decrease in the amount of unbound water, reducing inter-particle lubrication during flow [9]. Several authors have found that the presence of high proportions of swelling excipients, such as cellulose derivatives (>25% *w*/*w* Ac-Di-Sol), can lead to increased particle aggregation in the dispersion medium network [9,33,34,35]. This particle aggregation could have increased during the storage time, leading to a spatial expansion in which the particles enlarge omnidirectionally, thus increasing the friction of the semi-solid mass with the syringe walls and the nozzle walls. On the other hand, in the case of lactose (>25% *w*/*w*), the capacity to increase the binding between particles has also been observed by increasing the solid fraction of the dispersed particles [9,36]. As a final consequence, the presence of 50% *w*/*w* of these excipients (lactose + AcDiSol) increased the pressure required to reach a steady flow [9].

Several authors have determined that rapid viscosity recovery after the plunger compressive stress has ceased, as well as a low percentage of recoverable stress, are the ideal parameters for 3DP ink [9,20,28,37,38]. In all cases, after the cessation of a compressive force, the recorded pressure dropped rapidly (Figure 4) to a value where the flow of semi-solid formulation through the nozzle ceased. In the case of the syringe that was extruded successive times (S5), the percentage of recoverable stress decreased progressively. In fact, the accumulation of recoverable stress under successive extrusion cycles could lead to filament deformation and an uncontrolled extrusion process [9].

As established by Zidan and other authors, it is possible to determine the range of pressures with a steady flow of formulation through the nozzle from the extrudability profile [9,20,38]. Despite the important pressure differences required to achieve steady flow in each of the samples tested, once this pressure value was achieved, they showed very similar semi-solid flow through the nozzle as a function of time Q_weight, time_ and displacement Q_weight, displacement_ (mg/mm). However, after 48 h of controlled storage, there are slight variations in the extruded mass per mm of plunger displacement or time of application of the extrusion force, probably due to changes in the physical properties of the mass during storage time. The energy required to achieve the maximum pressure (AUC_1_) remains around 2.0·10^4^ kPa·s for print cartridges stored for less than 48 h; however, for longer storage times, this value increases with increasing storage time. The energy required to extrude one milligram of a semi-solid formulation during steady flow is expressed in Table 2 (AUC_2_/ total weight) and increased considerably as the storage time increased.

Young modulus shows the capacity of certain materials to resist elastic deformation. Therefore, the elastic modulus of a material increases with stiffness. Results obtained showed an increase during storage time or extrusion cycles after 24 h and a decrease in later periods of storage due to changes in internal structure, as previously mentioned [9,33,34,35].

By characterizing each of the semi-solid masses, it was possible to determine the optimum pressure range for each of the conditions. In this way, the range of pressures between the maximum applied pressure and the pressure of the steady flow was established as the limits to which the printing process would be under control.

#### 3.2.2. Printability of the Feedstock

According to several authors, “printability” refers to an ink’s capacity to achieve extrusion and maintain shape fidelity while printing with high accuracy. Printability is influenced by factors such as rheological and nozzle features before printing, design, slicing, g-code parameters such as pressure, temperature, and feed rate during printing, non-g-code parameters such as environmental conditions during printing, and post-printing parameters (drying techniques) [20,39].

**Figure 5:**
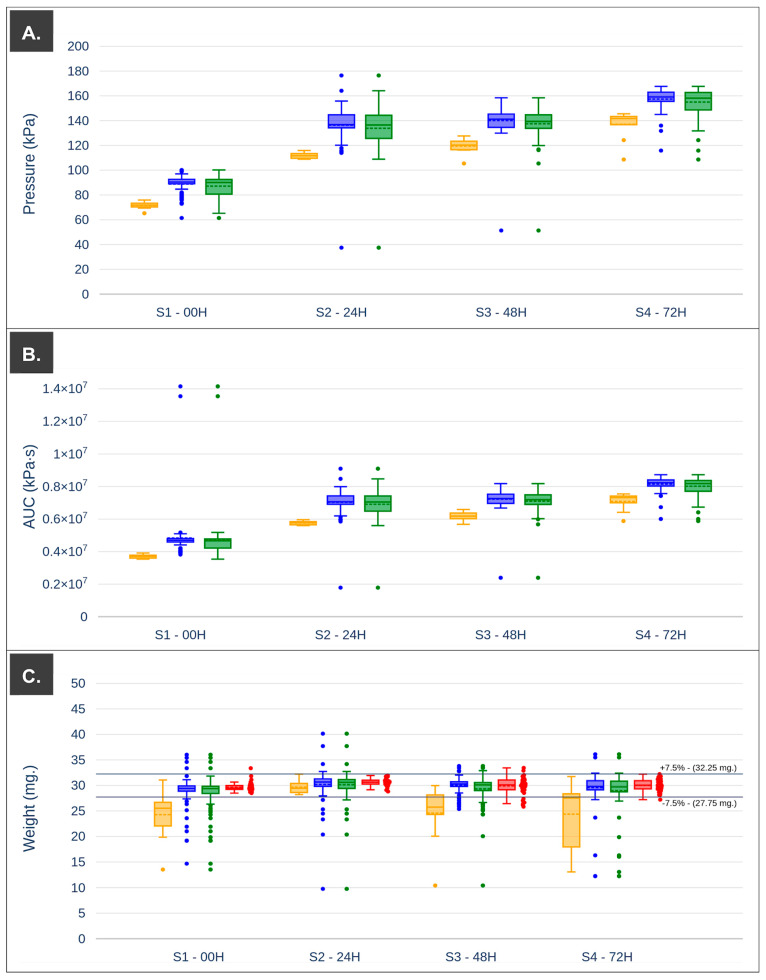
(**A**) Boxplot pressure with outliers. (**B**) Boxplot AUC (pressure vs. printing time) with outliers. (**C**) Boxplot weight with outliers and printlets validated by the algorithm (red). Data segmentation of the first 10 printlets (yellow—left), the next 90 printlets (blue—center), and whole printing process (green—right) was performed to highlight the differences.

According to the extrudability analysis, the 3D printing platform was set up to perform pre-extrusion pressurization of the printing ink at 70 kPa, and thus approach the beginning of the semi-solid mass flow. As expected, in all cases, the first 10 printlets (i = 1, …, 10) of each batch showed lower applied pressure and AUC values than the rest of the printlets (i = 11, …, 100) (Figure 5 and Table 3). These lower pressure values indicate that during this period of the printing process, semi-solid material was extruded with a non-steady flow resulting in higher variability of weights with standard deviations around 6 mg (Figure 5C and Table 3). In turn, the average weight values of the first 10 printlets obtained with each print cartridge (S1–S4) decreased as the storage time increased; this may have been due to the fact that the pressure required to reach a steady flow rate increased as the storage time increased, thus requiring the extrusion of more printlets to reach that state.

After the extrusion of a certain amount of material, the pressure necessary for steady flow was reached (Table 2). For example, in the case of print cartridge S1, the average applied pressure ranges from 71.6 kPa (i = 1…10) to 88.9 kPa (i = 11...100). This last value was in the middle, where there was steady flow through the nozzle (82.5 kPa–99.5 kPa) for this storage time, the same was observed for the rest of the cartridges studied (S2–S4). The variation of printlet weights obtained when the pressure had reached the pressure required for steady flow (i = 11…100) showed a lower standard deviation compared to the first 10 prepared. Differences in the outlier data points were observed for the applied pressure and the total energy required to produce a printlet, with less dispersion of the points within the established limits in the case of the AUC values and more clearly defined outliers. Based on the values of the average applied pressure (and its trend during processing), the energy used for each printlet, and data from computer vision, the printlets which weighed the same as the target weight (30 mg) were filtered. This subset of data was represented in the boxplot in red (Figure 5C); additionally, the established weight limits at 7.5% of the target weight were represented with solid black lines. Some values were above these limits for the print cartridge stored for 48 h, indicating that the algorithm will have to be refined in order to be cataloged more accurately in some circumstances. Considering these data, the first 10 printlets in each of the batches produced were set aside. The API content was shown to be close to 100% of DV for all storage times, as shown in Table 3, with a slight increase for the print cartridge stored for 72 h under controlled conditions. The reduction in the size of the printlets was approximately 45% in all cases, and shape fidelity was within acceptable values (97–103%).

**Figure 6:**
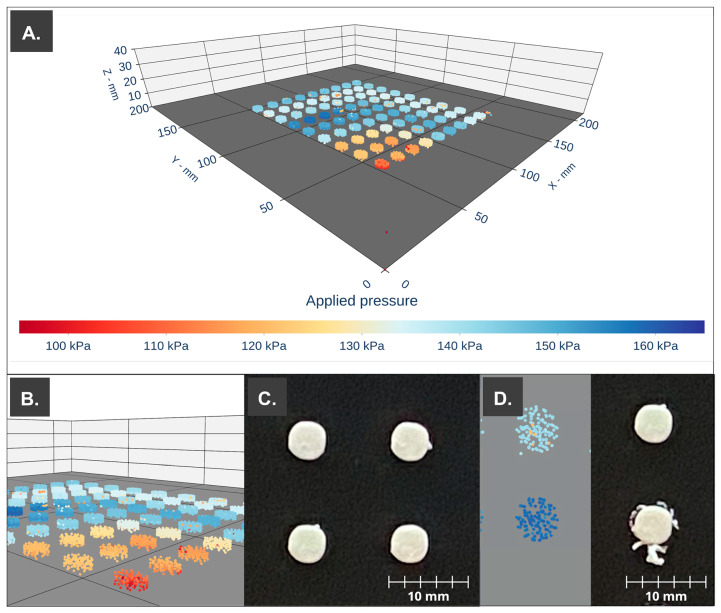
PAT apply to the 3D printing process of the print cartridge storage during 48 h (S3). (**A**) Three-dimensional scatter. (**B**) First 10 printlets printed with a lower applied pressure (red–orange). (**C**) Printlets (H2, H3, I2, and I3). (**D**) Normal printlet (top) vs. printlet during a detected clog (bottom), pressure point cloud of each printlet (left), and high-definition photography (right) (Printlets H2 and G2 (error)).

Despite the significant difference between the pressure required to achieve steady mass flow through the nozzle for each of the storage times, dosage forms with low mass variation and the required API content were obtained, indicating that it is possible to use the print cartridge after storage for up to 72 h after processing. As an example, Figure 6A shows the 3D scatter plot with the recorded pressure values located in the spatial coordinates. In the front, we can observe lower pressure values at the beginning of the steady flow, which for the printing ink (S3) is around 141 kPa, offering consistency with a lower weight and higher variability (24.38 mg ± 6.43 mg). Figure 6D shows the difference between a printlet performed with a controlled pressure value (150 kPa—light blue, upper) compared to a printlet during nozzle clogging (>160 kPa—dark blue, lower). The defects are clearly visible.

### 3.3. Stability Evaluation of Printlets

In Table 4, the results of stability studies are shown.

Printlets were stable, and API content remained between 95 and 105% during 28 days under accelerated conditions. The variability of % DV could be explained by the variability of the printing process itself and the analytical method, whose coefficient of variation was 4.7% [24,25]. In addition, a weight reduction of 6% has been observed during storage due to temperature.

### 3.4. Evaluation of the Ink and Process Capability to Print Different Doses

**Figure 7:**
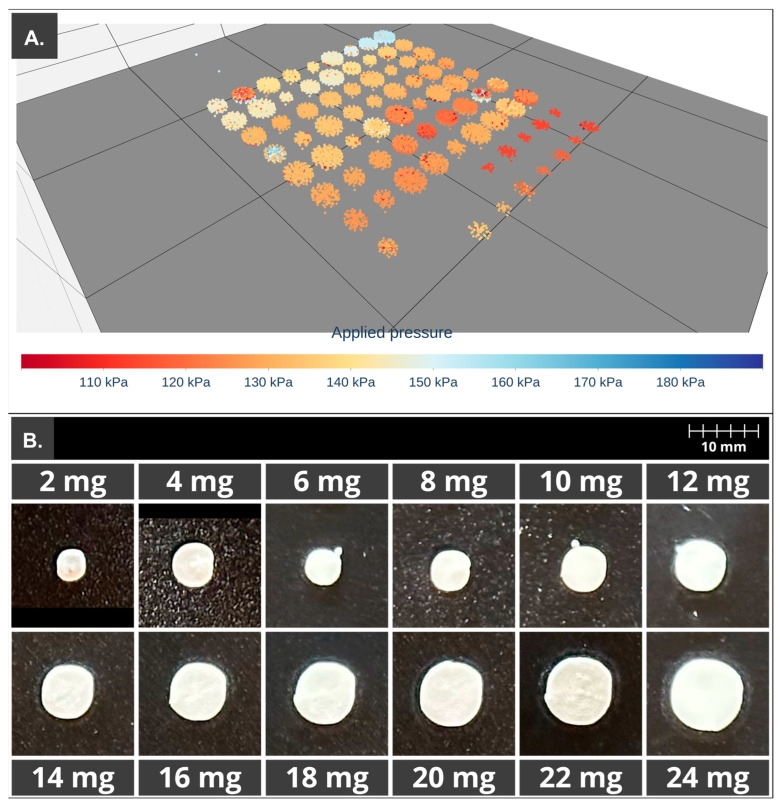
PAT apply to the 3D printing process of the print cartridge storage for 24 h. (**A**) Three-dimensional scatter, first 10 printlets printed with a lower applied pressure (red–orange). (**B**) Comparison of printlet size of 2–24 mg of HCT.

In order to achieve more stable values of AUC and pressure during the printing process, the print cartridge was kept for 24 h at 25 °C. The printlets were then produced using a g-code containing dosage units of different sizes in order to obtain different doses of API. In this case, the initial pressure set in the printer, at which the printing was started, was set to 130 kPa in order to ensure the steady flow pressure and thus reduce the weight variation in the first printlets as opposed to what happened in the print cartridges (S1–S4) previously mentioned. Figure 7A shows the 3D scatter plot with the recorded pressure values located in the spatial coordinates, and 6B shows the comparison of printlet sizes depending on API dose.

In Table 5, data from the validation of the printing ink are shown. The ANOVA of the linear regression confirmed the linearity between the expected and the real dose of HCT through rejection of the null hypothesis of deviation from linearity for a significance level of 0.05 (α = 0.05). The variation coefficient was 4.52%. The equation of the regression line was the area (μV·sec^−1^) = 1.02·C (mg); r^2^ = 0.987 from 6 to 24 mg. The representation of the residuals for each concentration showed a greater dispersion for those printlets with 2 and 4 mg of API; that is the reason why the ANOVA was performed starting with a higher concentration of 6 mg.

As can be seen in Table 5, percentages of DV for printlets with 2 and 4 mg of HCT are nowhere near the established limits, 92 and 93%, respectively. In the case of dosage forms with 6 mg of API, its average is 101.5%, but its standard deviation was high. These problems related to doses from 2 to 6 mg could be due to the reduced amount of mass, which must be extruded to obtain such doses, and the greater impact of errors during the 3D printing process, such as the presence of air in the formulation. When higher doses were elaborated, the % DV remained around 100%, with acceptable standard deviations lower than 10%.

Studied parameters such as shape fidelity showed values around 100% from 6 to 24 mg, meaning a good relationship between the theoretical diameter equivalent obtained from the g-code and the equivalent diameter from image analysis of the recently made printlets. This means a good buildability of the print cartridge and an optimum deposition of the extruded mass. However, when 2 and 4 mg were elaborated, the shape fidelity was below 95%, indicating a reduction in the dimensions of the printlet, which is related to a lower amount of mass extruded. In addition, size reduction was a little lower in the case of the lower doses in comparison to doses between 6 and 24 mg; this could be related to the fact that printlets with 2 and 4 mg were the last elaborated.

El Aita et al. did some comparable studies, but the dose of API was set based on the number of layers printed. In this study, Levetiracetam was used in API-elaborating dosage forms, with doses from 10 to 130 mg [1]. As mentioned before, our strategy considers the amount of extruded mass to obtain a certain dose and is, therefore, more flexible in comparison with the limitation of the amount of API per layer. In addition, printlets with lower doses, 6 mg instead of 10 mg, were obtained with acceptable quality attributes.

Therefore, according to these results, several doses could be elaborated in the same working plan, ensuring 100% of DV when the dose is equal to or higher than 6 mg. If a lower dose is needed, a new printing ink with a lower proportion of API should be used, as the amount deposited will be greater. 

## 4. Conclusions

An appropriate methodology for the rapid development of suitable SSE 3DP inks for pediatrics was established and evaluated. Firstly, the in situ extrudability analysis and several parameters allowed the detection of changes in the mechanical behavior of the masses, which must be considered to select the correct printing pressure after each of the storage times. In this sense, the determination of the pressure interval in which there was a steady flow of printing ink allowed us to establish the optimal printing pressure for printlets with the appropriate quality attributes, such as mass uniformity. Moreover, the energy required to extrude a quantity of one mg of semi-solid mass allowed the detection of important deviations in the value of the pressure applied during the printing process, thus detecting anomalies such as nozzle clogging or the inability of the system to extrude the mass due to the presence of air.

Secondly, the extrudability profile allowed the selection of the volume of semi-solid mass to be extruded and thus obtain the required doses. Moreover, the information provided made it possible to apply several PAT, such as the recording of the average pressure applied and the energy used in the production of each individual printlet, as well as computer vision to successfully use as control parameters during the printing of the dosage units. Then, through the use of the proposed Python algorithm, it was possible to detect the printlets that did not meet the CQA target weight, facilitating the automated control of the whole process.

The print cartridges were stable for up to 72 h after processing, and it was demonstrated that orodispersible printlets containing 6 mg to 24 mg of HCT can be produced using the same print cartridge during the same printing process with guaranteed content and chemical stability for at least 28 days.

Hence, the implementation of the proposed workflow for the development of new printing inks containing APIs will allow the optimization of feedstock material and human resources in pharmacy or hospital pharmacy services, thus speeding up their development and reducing costs.

## Figures and Tables

**Table 1:** Studied parameters.

Variable	Units	Description	Data Source
Start flow pressure (yield point)	kPa	Pressure value where the non-steady flow through the nozzle starts	Pressure–time plot
Max. applied pressure	kPa	Maximum applied pressure value recorded when the extrusion force is developed	Pressure–time plot
Steady flow pressure	kPa	Last time-sequence pressure value when the flow of semi-solid mass through the nozzle is constant	Pressure–time plot
Flow cessation pressure	kPa	Pressure value after the extrusion displacement of the plunger when the flow of semi-solid mass is interrupted	Pressure–time plot
Recoverable stress	%		$\frac{Flow\;Cessation\;Pressure}{Steady\;Flow\;Pressure}\cdot 100$
Q_weight, time_	mg·s^−1^	Flow of semi-solid mass through the nozzle as function of the test time	Weight–time plot
Q_weight, displacement_	mg·mm^−1^	Flow of semi-solid mass through the nozzle as function of the test displacement	Weight–distance plot
AUC_1_	kPa·s	Energy required to reach the steady flow of the semi-solid material through the nozzle	Pressure–time plot
AUC_2_	kPa·s	Energy used to extrude a certain amount of semi-solid material	Pressure–time plot
AUC_2_/total weight	kPa·s·mg^−1^	Energy used to extrude 1 mg of semi-solid material when the steady flow was reached	Pressure–time plot
Young’s modulus	kPa	Indicator of a material’s capacity to withstand length changes caused by longitudinal tension or compression.	Pressure–distance plot, slope of the linear part of the pressure–displacement curve

**Table 2:** Results for extrudability analysis of each print cartridge (S1–S5) and for the conditions previously established. SD: standard deviation; % DV: percentage of declared value.

Storage Time	0 h		24 h		48 h		72 h	
Print Cartridge	S1	S5	S2	S5	S3	S5	S4	S5
Start flow pressure (yield point) (kPa)	78.5	86.7	58.0	58.7	79.2	83.4	78.8	73.6
Max. applied pressure (kPa)	99.5	94.6	140.5	135.7	155.4	184.3	163.8	213.3
Steady flow pressure (kPa)	82.5	93.2	137.4	134.0	141.8	170.4	160.7	209.2
Flow cessation pressure (kPa)	47.6	58.4	80.4	78.5	89.3	98.4	107.3	110.9
Recoverable stress (%)	57.7	62.6	58.6	58.6	63.0	57.7	66.8	53.0
Q_weight, time_ (mg/s)	2.08	2.08	2.07	2.04	2.16	2.04	2.00	2.15
Q_weight, displacement_ (mg/mm)	415.3	415.3	414.0	408.5	432.6	408.4	400.3	430.3
AUC_1_ (10^4^ kPa·s)	2.16	2.17	2.39	2.07	3.98	4.71	4.59	5.07
AUC_2_ (10^4^ kPa·s)	4.12	3.92	5.78	6.09	4.31	4.94	4.91	6.64
AUC_2_/total weight (kPa·s·mg^−1^)	39.4	42.7	58.4	56.0	53.3	69.2	72.8	85.1
Young’s modulus (kPa)	52.8	60.3	90.2	92.5	51.0	81.6	47.2	66.4

**Table 3:** Results for the printing process control of each print cartridge (S1-S4) for the production of 100 printlets (10 mg) SD: standard deviation; % DV: percentage of declared value. * Calculated using theoretical volume (mm3) of extruded material required for the production of a 10 mg HCT printlet using the parameters calculated in the extrudability analysis section.

Storage Time		0 h	24 h	48 h	72 h
Print Cartridge		S1	S2	S3	S4
Applied pressure (kPa) (mean ± SD)	i = 1…10	71.6 ± 3.0	111.8 ± 2.5	119.5 ± 6.4	137.1 ± 11.8
	i = 11…100	88.8 ± 6.7	136.3 ± 16.1	140.1 ± 12.8	157.4 ± 8.6
	i = 1…100	87.1 ± 8.3	133.9 ± 17.0	137.5 ± 13.9	154.9 ± 11.2
Printlet weight (mg) (mean ± SD)	i = 1…10	30.1 ± 6.6	28.6 ± 6.3	24.4 ± 6.4	24.6 ± 5.8
	i = 11…100	29.0 ± 0.9	29.5 ± 2.0	30.0 ± 1.6	29.7 ± 3.2
	i = 1…100	29.1 ± 2.2	29.4 ± 2.8	29.4 ± 3.1	29.1 ± 4.0
Printlet estimated weight * (mg)		30.2	31.1	32.5	30.1
% DV, i = 11…100		99.6 ± 1.6	95.1 ± 0.6	99.6 ± 1.6	105.8 ± 0.2
Shape fidelity (mean ± SD, %), i = 11…100		97.8 ± 1.5	103.3 ± 1.5	101.5 ± 1.6	102.5 ± 2.9
Size reduction (mean ± SD, %), i = 11…100		44.5 ± 2.1	45.5 ± 1.5	44.3 ± 2.3	43.0 ± 2.8

**Table 4:** Results for stability evaluation of printlets (S1). SD: standard deviation, % DV: percentage of declared value, and % WR: percentage of weight reduction. All the results are expressed as mean ± standard deviation.

Time (Days)	% DV	% WR
0	99.6 ± 1.6	0.00 ± 0.0
3	102.9 ± 0.4	5.41 ± 0.2
7	99.2 ± 4.2	5.39 ± 0.3
14	104.6 ± 4.6	5.42 ± 0.2
21	104.2 ± 4.2	5.91 ± 0.2
28	99.3 ± 1.3	5.67 ± 0.2

**Table 5:** Results for the validation of printing ink. SD: standard deviation; % DV: percentage of declared value; and *n* = 3.

Dose (mg)	Weight (mean ± SD, mg)	Dose (mean ± SD, mg)	% DV (mean ± SD, %)	Shape Fidelity (mean ± SD, %)	Size Reduction (mean ± SD, %)
2.0	6.4 ± 2.6	2.6 ± 0.9	129.7 ± 46.2	92.6 ± 6.9	38.2 ± 2.1
4.0	9.6 ± 2.7	3.2 ± 0.8	79.2 ± 20.3	94.0 ± 4.3	39.3 ± 0.9
6.0	15.9 ± 2.6	6.0 ± 0.7	101.5 ± 10.4	103.0 ± 1.9	44.6 ± 2.2
8.0	21.3 ± 2.4	8.2 ± 0.5	102.6 ± 6.2	99.8 ± 1.6	42.5 ± 1.2
10.0	27.6 ± 2.3	10.4 ± 0.5	104.5 ± 5.2	99.5 ± 1.9	44.1 ± 1.2
12.0	32.9 ± 5.5	11.9 ± 0.8	98.7 ± 6.4	101.0 ± 2.5	44.3 ± 1.9
14.0	37.3 ± 4.9	14.7 ± 0.3	104.6 ± 2.1	100.1 ± 2.1	44.3 ± 1.8
16.0	44.6 ± 1.8	16.1 ± 0.6	100.6 ± 3.8	99.2 ± 0.3	43.2 ± 1.2
18.0	51.2 ± 3.5	19.0 ± 0.7	105.3 ± 3.8	100.3 ± 2.9	44.1 ± 1.4
20.0	55.7 ± 2.6	20.6 ± 1.1	102.7 ± 5.6	98.4 ± 1.9	43.7 ± 0.8
22.0	62.8 ± 2.0	22.4 ± 1.0	101.6 ± 4.3	99.5 ± 1.4	44.6 ± 0.5
24.0	67.7 ± 2.7	24.3 ± 1.1	101.4 ± 4.8	101.8 ± 2.4	43.8 ± 1.8

## Data Availability

Not applicable.
